# Shifts in Coding Properties and Maintenance of Information Transmission during Adaptation in Barrel Cortex

**DOI:** 10.1371/journal.pbio.0050019

**Published:** 2007-01-23

**Authors:** Miguel Maravall, Rasmus S Petersen, Adrienne L Fairhall, Ehsan Arabzadeh, Mathew E Diamond

**Affiliations:** 1 Instituto de Neurociencias de Alicante UMH-CSIC, Campus de San Juan, Sant Joan d'Alacant, Spain; 2 Cognitive Neuroscience Sector, Scuola Internazionale Superiore di Studi Avanzati—International School for Advanced Studies, Trieste, Italy; 3 Faculty of Life Sciences, University of Manchester, Manchester, United Kingdom; 4 Department of Physiology and Biophysics, University of Washington, Seattle, Washington, United States of America; Vanderbilt University, United States of America

## Abstract

Neuronal responses to ongoing stimulation in many systems change over time, or “adapt.” Despite the ubiquity of adaptation, its effects on the stimulus information carried by neurons are often unknown. Here we examine how adaptation affects sensory coding in barrel cortex. We used spike-triggered covariance analysis of single-neuron responses to continuous, rapidly varying vibrissa motion stimuli, recorded in anesthetized rats. Changes in stimulus statistics induced spike rate adaptation over hundreds of milliseconds. Vibrissa motion encoding changed with adaptation as follows. In every neuron that showed rate adaptation, the input–output tuning function scaled with the changes in stimulus distribution, allowing the neurons to maintain the quantity of information conveyed about stimulus features. A single neuron that did not show rate adaptation also lacked input–output rescaling and did not maintain information across changes in stimulus statistics. Therefore, in barrel cortex, rate adaptation occurs on a slow timescale relative to the features driving spikes and is associated with gain rescaling matched to the stimulus distribution. Our results suggest that adaptation enhances tactile representations in primary somatosensory cortex, where they could directly influence perceptual decisions.

## Author Summary

Neuronal responses to continued stimulation change over time, or “adapt.” Adaptation can be crucial to our brain's ability to successfully represent the environment: for example, when we move from a dim to a bright scene adaptation adjusts neurons' response to a given light intensity, enabling them to be maximally sensitive to the current range of stimulus variations. We analyzed how adaptation affects sensory coding in the somatosensory “barrel” cortex of the rat, which represents objects touched by the rat's whiskers, or vibrissae. Whiskers endow these nocturnal animals with impressive discrimination abilities: a rat can discern differences in texture as fine as we can distinguish using our fingertips. Neurons in the somatosensory cortex represent whisker vibrations by responding to “kinetic features,” particularly velocity fluctuations. We recorded responses of barrel cortex neurons to carefully controlled whisker motion and slowly varied the overall characteristics of the motion to provide a changing stimulus “context.” We found that stimulus–response relationships change in a particular way: the “tuning functions” that predict a neuron's response to fluctuations in whisker motion rescale according to the current stimulus distribution. The rescaling is just enough to maintain the information conveyed by the response about the stimulus.

## Abstract 3

Cortical neurons adapt their responses to changes in the input statistics of a stimulus, suggesting adaptation enhances stimulus discrimination and perception.

## Introduction

Adaptation is the accommodation of neuronal responses to an ongoing stimulus [1,2]. In the anesthetized rat primary somatosensory “barrel” cortex (BC), it is established that neuronal responses adapt robustly to repetitive whisker stimulation [3–11].

Although adaptation is observed almost universally across species and sensory modalities, in most cases its functional effects and underlying mechanisms have not been established. In several instances in the sensory periphery, adaptation helps neurons solve the fundamental problem of encoding signals that vary over a wide range compared to the range of responses available to the neuron. In these instances, adaptation involves shifts in the neuronal input–output relationship (tuning curve) following changes in the stimulus statistical distribution [12–16]. The shifts cause the range of neuronal responses to match the statistical distribution of the stimulus, thus optimizing information transmission [15–24]. Adaptation can occur to the stimulus distribution's mean, to its variance, and to other statistical properties [15,23–26], such as the correlations specific to natural stimuli [27]. In any system where responses to ongoing stimulation vary over time, it is important to understand whether adaptation constitutes a stimulus-specific change in coding.

Quantitative study of the role of adaptation in information transmission in sensory cortex is of particular interest for two reasons. First, the excitability of cortical neurons is strongly modulated by central factors other than sensory environment (e.g., [28–32]). It needs to be ascertained whether adaptation can enhance information transmission even in the face of large nonsensory inputs. Second, cortical activity is likely to be closely related to the sensory experience of the animal. Consequently, if cortical adaptation entails adjustments in neuronal coding, it can lead to a sharpening of discriminative capacities [27]. Motivated by this possibility, here we examined the effects of adaptation on vibrissa motion encoding. Whisker motion across a textured surface [33] induces vibrations with frequencies up to ∼200 Hz. The vibration associated with any texture is characterized by rapid, irregular, intermittent variations in velocity. In some cases, the vibrations evoked by different textures differ markedly in mean velocity [33,34]. Under these conditions, BC neurons could represent texture by encoding mean velocity by the firing rate averaged across a stimulus presentation [35]. However, textures with similar overall roughness and mean velocity can be discriminated only by the specific sequence of vibrations along the whisker sweep. In that case, firing rate by itself is not adequate to discriminate between textures; rather, the precise “kinetic signature” must also be encoded [35]. Thus, any adaptive mechanism that optimizes the representation of fine kinetic features could improve discrimination between textures.

To test whether adaptation may facilitate discriminations that depend on a precise representation of whisker kinetics, we applied stochastic, continuously changing stimuli distributed as a Gaussian in displacement and velocity. The Gaussian's variance in position and velocity switched back and forth between two set values, altering the parameters of the distributions—the statistical “context”—within which individual stimulus values were delivered. We asked whether neurons used fixed input–output tuning functions to encode individual stimulus values or, alternatively, whether they developed stimulus-specific functions that shifted according to the statistics of the ongoing distribution.

## Results

### Adaptive Responses to Switching Variance of Noise Stimuli

**Figure 1:**
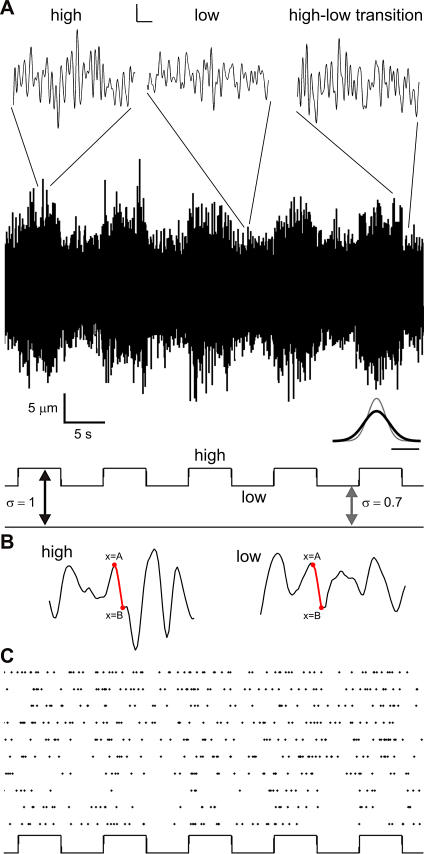
Adaptive Responses to Stimuli with Switching Variance (A) Vibrissa motion waveforms (central plot) were made by drawing instantaneous displacement values from a Gaussian distribution whose variance was switched every 5 s. The ratio of low to high variance was 0.49. The bottom plot shows the “mask” used to implement the switch in variance. Transitions were smoothed over 10 ms; stimulus values were smoothed over ∼5 ms and were white noise in frequency up to ∼210 Hz (see magnified plots at top; scale bars of magnified plots, vertical, 5 μm; horizontal, 25 ms). The inset plot shows the distributions' relative widths, where the *x*-axis is position and the *y*-axis is the probability density (scale bar, 20 μm). (B) A whisker displacement from *x* = A to *x* = B can occur against a context of high-variance (left) or low-variance stimuli (right). (C) Spike times from a single neuron's response over ten successive stimulus cycles. Stimulus values did not repeat, so the spikes were not temporally aligned on successive cycles.

We analyzed how adaptation affects the encoding of whisker motion in BC. Continuous, filtered white-noise (hereafter referred to simply as “noise”) stimuli were applied to the principal whisker and surrounding whiskers of recorded neurons. Motion varied on two timescales. First, instantaneous whisker position and velocity varied on a timescale of ∼5 ms. Second, the variance of the randomly fluctuating whisker deflections switched between two values at fixed intervals of 5 s duration (Figure 1A). The problem of interest was whether neuronal encoding of stimulus fluctuations on short timescales (up to tens of milliseconds) changed according to the longer-timescale statistical context within which fluctuations were delivered. Statistical context, in our experiments, was defined by the variance. For example, how is a specific stimulus event—whisker movement from position A to position B—represented when that event occurs against a background of large amplitude, fast movements as compared to a background of smaller amplitude, slower movements (Figure 1B)?

We examined response dynamics in a dataset of single layer 4 neurons that showed stable responses to noise stimulation (criteria for inclusion in the dataset are discussed in Materials and Methods). The dataset comprised 34 single neurons and multineuron clusters, of which ten single neurons fulfilled all selection criteria for spike-triggered covariance (STC) analysis (see below). For these neurons, average firing rates during stimulation were significantly (around six times) larger than before stimulus onset. Moreover, the rates were markedly higher in the high-variance epoch compared to the low-variance epoch (Figure 1C). Thus the neurons were both responsive to the noise stimulus and sensitive to the changes in stimulus statistics.

**Figure 2:**
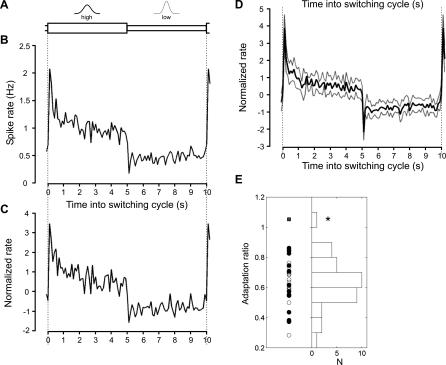
Adaptive Modulation of Spike Rate (A) Representation of stimulus distributions and switches during a cycle. (B and C) Behavior of the neuron shown in Figure 1B. Bin size, 100 ms. Dashed lines at 0 and 10 s show correspondence with start and end times of cycle. (B) Absolute spike rate averaged over switching cycles (1,080 repetitions). (C) Spike rates normalized by the total number of spikes in each 10-s cycle and then averaged over cycles, eliminating variations in absolute rate (spike count). Finally, the averaged rate was normalized by the spike rate standard deviation over the cycle, and its mean over the cycle was subtracted, giving a specific measurement of rate modulation. (D) Rate modulation in the normalized units of (C), pooled over all adapting single neurons (*n* = 9). Black, population average; gray, plus/minus population standard deviation. Rate modulation was robust and occurred over a similar timescale across the population. (E) Adaptation ratios. The firing rate at steady state (binned 4–5 s after each upwards switch in stimulus variance) was divided by the rate immediately after switching to high variance (binned 0–100 ms after the switch). Left: data points. Filled gray square, nonadapting neuron (*n* = 1); filled black circles, adapting single neurons (*n* = 13); open circles, adapting multineuron clusters (*n* = 20). Right: histogram of adaptation ratios for all recordings shown on the left side. Only a single cortical neuron showed nonadapting behavior. The asterisk denotes that this neuron's adaptation ratio was significantly different from that of the rest of the population (*p* < 0.001) (after Lilliefors test for normality on the rest of the population's distribution).

We tested neurons for adaptation as follows. Responses were quantified by measuring how firing rate varied during the course of the switching cycle (Figure 2). Neurons typically showed prominent adaptation in firing rate. Mean firing rate sharply increased when the distribution of stimulus positions and velocities switched from low to high variance and then slowly adapted, or declined, as high-variance stimulation continued. After switching back to low variance, firing probability decreased sharply and then recovered somewhat during the course of the low-variance epoch (Figure 2B and 2C). Of the ten highly stable single neurons selected for full analysis, nine displayed clear adaptive dynamics (Figure 2D). In total, 33 out of the 34 single neurons and multineuron clusters in the dataset showed adaptation (Figure 2E) (the nonadapting neuron is discussed below).

### Time Courses of Adaptation

The time course of rate adaptation was on the order of hundreds of milliseconds for all the adapting single neurons (Figure 2D) (280 ± 180 ms, range 150–550 ms, *n* = 9). This presents an interesting contrast to the timescales over which BC neurons encode individual stimulus parameters. Upon whisker deflection with steps, ramp-and-hold pulses, or sinusoidal waveforms, BC neurons have a transient response to stimulus onset during which their firing rate robustly encodes whisker velocity [3,36,37]. The firing rate then typically shows a rapid decline (within 10 ms of the response onset) (e.g., [37]) to a low level of tonic firing, although adaptive decay of responses can continue over a longer timescale [8,11]. With noise stimuli, the time course of rate adaptation was an order of magnitude longer than the time course associated with stimulus-feature encoding. This suggests that the responses of BC neurons did not depend only on local stimulus fluctuations, but also depended on changes in the stimulus distribution.

### Adaptive Changes in Coding Scheme

Although adaptive changes in firing rate were present in the great majority of recorded single neurons, this need not indicate modulation of stimulus–response relationships (e.g., [38,39]). For instance, the velocity sensitivity of BC neurons can account for their strong response to the ramping phase of a deflection and their “adapting” response to a maintained deflection [36]. Further, decreased responses to repetitive stimulation can be due to fatigue of neuronal and synaptic processes. We investigated whether the rate adaptation we found was of this type, or whether it was associated with a true modulation of the stimulus–response relationship.

One property common to noise stimuli and naturalistic texture stimuli is that the timescale over which the stimulus can vary (here, ∼5 ms) is much shorter than typical BC inter-spike intervals or neuronal integration times [33]. Because of this, the output of BC neurons represents a temporally filtered version of the stimulus, characterized by a reduced set of properties, or features. For BC neurons tested with naturalistic stimuli, relatively simple stimulus features such as velocity account for much of the response [33], in common with other well-characterized neurons that detect motion [22]. In general, understanding a stimulus–response relationship requires identifying the set of stimulus features to which a neuron is sensitive and estimating the input–output functions, or tuning curves, that describe how features modulate the neuron's firing probability. A powerful way to do this is STC analysis [22,40]. As detailed in Materials and Methods and in Protocol S1, this technique first identifies the relevant set of features. Having extracted these features, it then computes the generally nonlinear input–output function governing the neuron's responses to them. The identified features and input–output function constitute a “linear–nonlinear” characterization of the neuron's stimulus–response relationship (reviewed in [41,42]).

To test how adaptation affects coding of whisker stimuli, we carried out STC analyses once response rates had reached steady state (2–5 s after each switch). We analyzed high- and low-variance stimulus periods separately to determine which aspects of stimulus coding were altered by adaptation.

**Figure 3:**
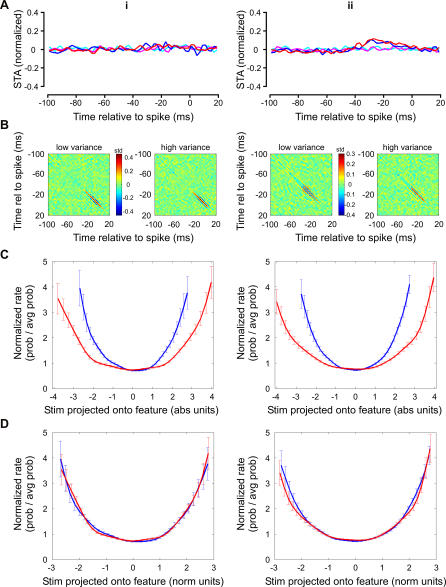
STC Analysis Columns (i) and (ii) show results for two single neurons recorded in different animals. All results were computed for 2–5 s after stimulus switches. (A) STA and random averages normalized by the corresponding stimulus standard deviation. Cyan*,* random (prior distribution) average over low-variance periods; blue, STA over low-variance periods; magenta, random average over high-variance periods; red, STA over high-variance periods. Neuron (i) was typical in having an STA without significant structure; neuron (ii) had the largest STA among adapting neurons, although still small. Neither neuron showed adaptive changes in STA shape. (B) Covariance difference matrices, showing the difference between the spike-triggered and prior covariance matrices, for low- and high-variance periods, shown in units of the corresponding standard deviation (color bar). (C) Nonlinear input–output relationships for low-variance (blue) and high-variance (red) periods. Modulations of spike rate were normalized by average rate and plotted against the stimulus projection onto a significant feature extracted from the covariance difference matrix. Stimulus projections were plotted in absolute units proportional to real wafer displacement (equal in value to the low standard deviation). Error bars are the standard deviation from 30 repetitions of the estimation procedure. (D) Replotted input–output relationships with inputs normalized by their corresponding standard deviation. Error bars as in (C). For both neurons adaptation involved a rescaling of input–output relationships according to input range.

Figure 3 shows results for two different neurons with typical responses. First, we observed that spike-triggered averages (STAs) were either negligible (Figure 3A, neuron i) or small in amplitude compared to the stimulus standard deviation (Figure 3A, neuron ii), and therefore could not account for the neurons' responses. This may happen if the features driving the neuron have symmetries such that their mean is zero (Figure S1, panels A3 and C2; explained in Protocol S1). Use of the STC method uncovered the relevant stimulus features. Meaningful structure in the stimulus distribution that evoked responses was captured by a covariance difference matrix **Ĉ** (see Materials and Methods, Protocol S1, and Figure S1). **Ĉ** matrices had clear “hot spots” marking the times when the spike-triggered stimulus distribution differed from the overall “prior” stimulus distribution. These structures included a positive diagonal (variance) band extending back −30 or −40 ms relative to spike time, weaker negative side bands, and sometimes a weak negative diagonal “tail” extending from the end of the positive band back to approximately −80 ms (Figure 3B; note the similarity to the model covariance matrix in Figure S1, panel D3). The duration of these structures defines the timescales over which neurons were sensitive to individual stimulus fluctuations (see above). Apart from the change in the distribution's overall scale, **Ĉ** matrices had the same structure for both stimulus distributions (Figure 3B): when **Ĉ** was given in units normalized to the corresponding stimulus distribution's standard deviation, changes in stimulus scale did not appreciably change matrix structure. Neurons were selective to between one and six (usually two or more) stimulus features, of which the most significant were usually stimulus velocity and acceleration smoothed over tens of milliseconds (see Figure S1). Similar results have been observed in other studies (R. S. P. and M. A. Montemurro, unpublished data). As implied by the results for **Ĉ** *,* switching the stimulus distribution caused no qualitative changes in the features: independently of the ongoing statistical “context,” neurons were sensitive to the same kind of physical events.

The next step was to estimate the input–output relationships that describe how the neurons were driven by the presence of each physical feature. If a neuron is responsive to a given feature, its response to a stimulus segment depends on the magnitude of the stimulus projection onto that feature (i.e., how much the stimulus resembles the feature) (see Figure S1). For instance, if a neuron is sensitive purely to instantaneous velocity, its response is determined by the difference in position at successive time points. The more similar the stimulus is to this “derivative” waveform, the greater the response will be. The input–output function captures the neuron's sensitivity to the feature. We estimated each neuron's input–output function by plotting modulations in firing probability as a function of the stimulus projected onto each of the features constituting the neuron's receptive field (Figure 3C and 3D; see Materials and Methods). The input–output functions' symmetry with respect to the sign of the projection implies that neurons were sensitive to features' absolute value (e.g., absolute velocity [equivalent to speed]).

We plotted input–output functions against stimulus projections measured in units proportional to true whisker displacements. Figure 3C shows the result for the same two neurons represented in Figure 3A and 3B. In the absolute units of Figure 3C, the scales of input–output functions depended on the distribution's scale. During high-variance periods (red curves), curves were more stretched along the input (stimulus) axis: a larger change in input was required to cause a given proportional change in firing probability than during low-variance periods (blue curves). This suggested that neurons rescaled their sensitivity, or gain, according to the input distribution. To determine the amount of rescaling, we replotted the curves, normalizing the input scale by its standard deviation [16,22]. Now, the difference between the input–output functions disappeared (Figure 3D). This result applied not just to the input–output curve computed for the most important feature in each neuron's receptive field (usually the velocity), but to input–output curves computed for all features.

The central result of this analysis is that the input–output curves for high- and low-variance periods (Figure 3C) scaled with the stimulus standard deviation. Normalized for stimulus standard deviation, input–output curves conserved their shape across changes in input distribution. Therefore, adaptation rescaled neuronal input–output relationships exactly in proportion to the stimulus distribution's standard deviation.

### Absence of Rate Adaptation and Corresponding Absence of Input–Output Rescaling

**Figure 4:**
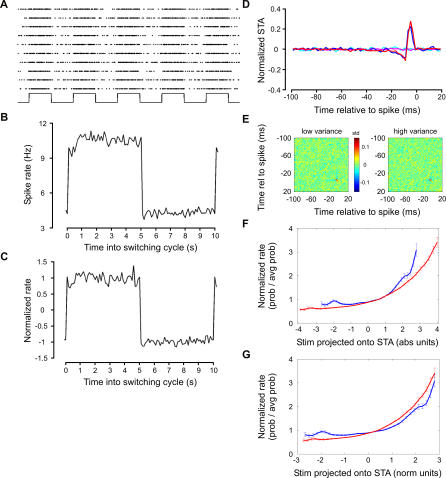
Firing Rate and STC of a Neuron That Lacked Adaptation (A) Responses to ten successive stimulus cycles (bottom plot, variance mask). (B) Absolute rate modulation during switching cycles. Spiking rate remained high throughout high-variance periods and low throughout low-variance periods. Bin size, 100 ms; responses averaged over 1,040 repetitions. (C) Normalized rate modulation computed as for Figure 2B. (D–F) Covariance analysis computed 2–5 s after variance switches. (D) Spike-triggered and random averages. Cyan*,* random average, low-variance periods; blue, STA, low-variance periods; magenta, random average, high-variance periods; red, STA, high-variance periods. There was no change in STA shape. (E) Covariance difference matrices for low- and high-variance periods, normalized by the local standard deviation (color bar). There was very little structure in the covariance difference matrices, and no visible change after variance switches. (F) Nonlinear input–output functions plotted against the stimulus projection onto the STA measured in absolute units as in Figure 3C: low-variance (blue) and high-variance (red) periods. Error bars as in Figure 3C. The neuron was more sensitive during high-variance stimulation. (G) Nonlinear input–output functions normalized by the local standard deviation as in Figure 3D. Input–output relationships did not fully rescale.

For one neuron in the dataset, firing rate changed dramatically at each switch in stimulus variance but did not adapt within the variance epochs (Figure 4A–4C). This neuron was unique by other criteria. First, it had a high average firing rate across both stimulus conditions (7.5 spikes/s) compared to other neurons (1.2±0.3 spikes/s). Second, it had a large, sharp (<20 ms) STA (Figure 4D), while its **Ĉ** matrix showed little structure (Figure 4E); the STA was, therefore, the stimulus feature that contributed most significantly to its receptive field (Figure S2). In ongoing work (M. M. and R. S. P., unpublished data), we have found that thalamic neurons often have a significant STA and lack adaptation; therefore, the nonadapting neuron described here may have been a thalamic fiber or a fast-spiking cortical neuron (thalamic inputs can dominate the responses of fast-spiking neurons [43–45]). STC analysis showed that the neuron's input–output relationship did not rescale (Figure 4F and 4G). These data demonstrate that nonadapting sensory responses, albeit rare, can be present in BC. Although rate adaptation and input–output rescaling could occur through separate processes [16,24], the absence of both of them in the same neuron is consistent with the idea that they are associated.

### Adaptation and the Maintenance of Stimulus Information

One way to quantify the effects of adaptation is to estimate the information carried by a neuron's responses about the stimulus. Because a nonadapting neuron does not change its coding properties, one would expect it to be better tuned to the dynamic range of one particular stimulus distribution, and convey more information about that distribution than about distributions with a mismatched range. Conversely, if a neuron remains sensitive to the same features following adaptation, but rescales its input–output relationships so that they preserve their shape, an intriguing possibility is that the amount of rescaling may be of precisely the amount necessary to conserve information transmission under different conditions [22].

**Figure 5:**
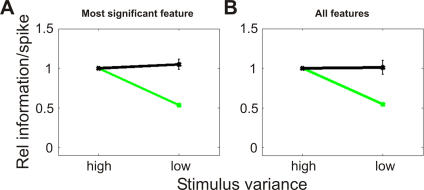
Mutual Information per Spike between Responses and Stimulus Features during Adaptation Black, average over adapting neurons (*n* = 8); green, nonadapting neuron from Figure 4. Error bars show standard error of mean. (A) Information conveyed about the most significant feature. During high-variance periods, absolute information was 0.12 ± 0.02 bits per spike; computed over adapting and nonadapting neurons, *n* = 9. (B) Estimate of summed information about all significant features. During high-variance periods, absolute summed information was 0.29 ± 0.07 bits per spike, *n* = 9. Neurons with adaptive responses transmitted constant quantities of information per spike in the face of changes in stimulus distribution, but for the neuron with no adaptation, mutual information decreased when the stimulus distribution changed.

We therefore estimated the information conveyed by individual spikes about stimulus features [38,46] in both high- and low-variance conditions, once responses had reached a steady state 2–5 s after stimulus switches. The first step was to estimate information about the stimulus feature that contributed most significantly to each neuron's receptive field. To compare neurons, information per spike was normalized by its high-variance value. For the nonadapting neuron, information about the STA in the low-variance periods decreased to 0.53 times its value in the high-variance periods (Figure 5A). In contrast, for adapting neurons, information conveyed about the most significant feature was maintained: in low-variance periods its value was 1.05 ± 0.06 relative to that in high-variance periods (*n* = 8) (Figure 5A). The next step was to analyze the summed information about all significant stimulus features (between one and six features per neuron, usually two or more). This is equal to the total information per spike conveyed about the full receptive field provided that all features contribute independently (see Materials and Methods). For the nonadapting neuron, the summed information again decreased strongly during low-variance periods, to 0.54 times that for high-variance periods. In contrast, adapting neurons maintained the summed information: the low-variance to high-variance ratio was 1.01 ± 0.09 (*n* = 8) (Figure 5B). Adaptive coding thus caused neurons to conserve the information per spike conveyed about stimulus features, in the face of changes in stimulus statistics.

### Slow Adaptation and Gain Rescaling as a Reflection of Changes in Coding Parameters

**Figure 6:**
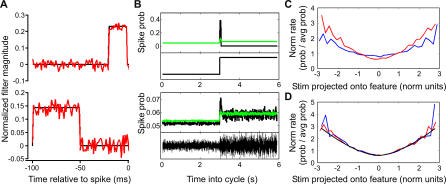
Simulations of Adaptive Responses Using Fixed Features (A) Stimulus segments were projected onto two orthogonal features (black): a sharper excitatory filter (top) and a longer suppressive filter (bottom). The projected stimulus was fed into a nonlinear function used to compute the probability of spiking at time *t* = 0. In red, filters recovered by STC analysis conducted on responses collected over a simulated 800 s. (B) Spike rate modulation upon onset of a square pulse stimulus (top) and upon an increase in white-noise stimulus variance (bottom). Responses averaged over 300 repetitions. Black and green curves show responses for simulations performed with different sets of parameters for the spiking nonlinearity. In all cases tested, adaptation to white noise was less pronounced than adaptation to the square pulse, but the timescale of adaptation was never longer than the duration of the longest (suppressive) filter. (C) Estimated normalized input–output functions from the STC analysis for low- (blue) and high-variance (red) periods: stimulus-dependent modulations of spike probability plotted as in Figure 3D. Note that the functions' zero-crossing level did change. This is expected because the way in which firing probability is modulated by the stimulus projection onto the feature of interest will differ across high- and low-variance periods, even for a nonadapting model. (D) Estimated normalized input–output functions represented as in (C), for stimuli divided by their distributions' standard deviations. The true function appears in black.

Several mechanisms can produce the appearance of adaptive changes in coding without true changes in input–output functions [47]. One possibility derives from the fact that neurons can be sensitive to two types of stimulus features, “excitatory” and “suppressive” [48]. For an excitatory feature, the firing probability increases with stimulus similarity to the feature (i.e., as a function of the magnitude of the stimulus projection onto the feature); for a suppressive feature, firing probability decreases with stimulus similarity to the feature [48]. Thus when an ongoing stimulus matches a suppressive feature, the firing rate can decrease even without concomitant changes in input−output function. We constructed and analyzed a model neuron to see whether slow firing rate adaptation and gain rescaling like that observed in BC neurons could occur even without real changes in the neuronal tuning curve, simply via the presence of suppressive features. The simulated neuron was sensitive to one excitatory feature and one (orthogonal) suppressive feature (Figure 6A). As in our linear−nonlinear representation of experimental neurons, firing probability depended on the amount of overlap, *k,* between the stimulus and each feature comprising the neuron's receptive field. Spikes were generated through a nonlinear threshold function that gave the dependence of firing probability, *p,* on *k:* 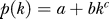 with free parameters *a, b,* and *c,* a generic functional dependence that fits BC neurons (the specific choice of *a, b,* and *c* did not affect the conclusions below) (Figures 3C and 4F) (R. S. P. and M. A. Montemurro, unpublished data). In simulations run with a range of values for the free parameters, firing rates decreased less after upwards switches in white-noise stimulus variance than after the onset of a maintained square step (Figure 6B), just as for real neurons. However, rate adaptation in the model never occurred over a time course longer than the suppressive feature's duration. Other simple models using a single excitatory filter sensitive only to the stimulus “velocity” showed the same behavior, in that the adaptation timescale equaled that of the longest-duration feature. This contrasted with the real neurons, where the timescale of rate adaptation was much longer than that of feature encoding.

We also used the model to analyze whether the rescaling of neuronal sensitivity observed in our experiments could arise as an artifact, without genuine changes in the input–output function's gain. After running each simulation, we recovered the model's filters (Figure 6A) and the input–output relationship for the excitatory feature and compared them across high- and low-variance stimulation periods just as we did for real neurons (Figure 3C). Over a range of stimulus variances and of functional dependences of *p*(*k*), there was never rescaling of the derived input–output function (Figure 6C). Gain rescaling with full overlap of input–output functions (i.e., the disappearance of the difference between input–output functions [Figure 6D]) occurred only when input rescaling was explicitly incorporated into the model (i.e., when the spike generator's input was not the raw stimulus but the stimulus normalized by its standard deviation).

In conclusion, these simulations argue against the possibility that the adaptive rescaling of neuronal sensitivity seen in our experiments could be accounted for without true shifts in coding. Taken together, our results suggest that, for BC neurons well captured by STC analysis, gain rescaling probably arises from modulation of neuronal coding mechanisms. The modulation is dependent on stimulus distribution and has the effect of maintaining information transmission about the stimulus features represented by the neuron.

## Discussion

These experiments demonstrate that adaptation in BC involves a change in neuronal tuning curves and serves to maintain the information carried about stimulus features in the face of changes in input statistics. Adaptation to stimulus statistics can enhance the information content of responses in the central auditory and visual pathways [23,27]. Because neuronal spiking in primary somatosensory cortex may directly influence sensory decisions [49,50], our findings support the idea that adaptation plays the role of enhancing stimulus discriminability.

The whiskers received the stimulus “passively,” unlike the “active” stimulation achieved by muscle-contraction-induced whisker sweeping [51,52]. Whisker “motion,” in our terminology, is a movement applied to the whisker, not a movement originating in muscle contraction. However, it is important to recognize that the same neuronal processing properties uncovered by randomized (“noisy”) whisker movements are likely to operate under more natural conditions. In earlier work in BC, neuronal firing probabilities to velocity events during a Gaussian noise stimulus accurately predicted the response of the same neurons to the highly structured velocity profiles associated with whisker movement across textures [33]. In the present work, input–output functions and their adaptation to stimulus statistics were derived from noise stimuli. Because adaptation of input–output functions is the outcome of fundamental processing properties of neurons and the circuits in which they reside, it is reasonable to expect that similar forms of adaptation hold for natural stimuli as for noise stimuli.

### STC Analysis of Changes in Input–Output Functions

The present work characterizes BC sensory coding by STC analysis, a generalization of reverse correlation [53,54]. We selected the STC method to study adaptation for two reasons. First, the negligible structure in the STA (Figure 3A) meant that a higher-order method was necessary to capture response properties. Second, the method provided a direct quantification of neuronal input–output functions, a coding property that we expected would be affected by changes in stimulus statistics. Recent applications of STC include the fly H1 motion-sensitive neuron [22,40,42], retinal ganglion cells [55,56], cat V1 complex cells [57,58], primate V1 simple and complex cells [48,59], and nucleus magnocellularis cells in the avian auditory brainstem [39], as well as models of spiking neurons [38,60,61].

Changes in input–output gain can arise even in simple, general neuronal models because of interactions between the stimulus distribution scale and the nonlinearities inherent to spike generation [47,60–63]. Although it is not possible to rule out a contribution of those generic nonlinear effects to the adaptation we observed, we do not believe they account for our findings. Any input–output gain rescaling resulting from the generic nonlinear threshold nature of spike generation should be similar across spiking neurons. But the response to stimulus switching was not uniform in our dataset: one neuron did not show firing rate adaptation, and it also lacked input–output gain rescaling; nine other neurons adapted and underwent rescaling of input–output functions. In addition, in simulated neurons, nonlinear threshold effects did not produce slow adaptation on the timescale of that observed in the nine studied adapting neurons. The simulated neurons also did not show rescaling of input–output functions: the absence of a slow timescale for rate adaptation was associated with the absence of adaptive changes in coding (see [47,64]).

### Adaptation to Stimulus Statistics: Mean Versus Variance

All neurons but one were sensitive primarily to whisker speed (absolute value of velocity). Since the mean absolute velocity was proportional to the velocity standard deviation, it is possible that neurons adapted to the absolute velocity mean rather than to the velocity standard deviation. A likely substrate for responsiveness to absolute velocity is rectification through the whisker synaptic pathway: neurons may receive net depolarizing inputs whatever the sign of the vibrissa deflection. If so, the net synaptic input onto BC neurons could be greater during high-variance periods, effectively increasing the stimulus distribution's mean. With the broadband Gaussian stimuli necessary for STC analysis these stimulus properties cannot be disambiguated, since both vary in proportion to the scale of the distribution of whisker displacements (see Protocol S1). Neuronal adaptation to one feature may constitute simultaneous adaptation to the other.

### Maintenance Versus Maximization of Information in BC

An important effect of adaptation on coding in the sensory periphery can be to optimize information transmission by rescaling input–output relationships to match the input distribution. Adaptation is clearly necessary when the full dynamic range of inputs is orders of magnitude larger than that of the receptor neurons (e.g., the natural distribution of light intensities): the absence of adaptation would seriously constrain the ability to represent the environment. The present experiments involve a more subtle form of adaptation inasmuch as the stimulus input range did not span the neuronal output dynamic range: the measured input–output curves did not saturate at steady state (Figure 3C and 3D). Even so, adaptation enabled neurons to maintain information transmission during changes in the stimulus distribution.

Indeed, while visual stimuli are generated by external sources whose luminance variations extend over many orders of magnitude, natural whisker stimuli are constrained by the limits of the vibrissa muscle contractions that generate the stimuli. Thus, neuronal input–output modulation in BC probably does not reflect constraints on neuronal dynamic range. Rather, adaptation might reflect a balance between information maintenance, the ability to accommodate internal brain-state modulations, and homeostatic regulation caused partly by the energy cost of spiking.

### Adaptation and Texture Discrimination

Could adaptation help BC neurons encode texture identity? As vibrissae sweep across a texture, the firing rates of BC neurons over periods of tens of milliseconds encode the whisker vibration's equivalent noise level, a generalization of time-averaged velocity [33]. However, different textures can induce similar equivalent noise levels or mean speeds: their identity is then transmitted by the vibration's detailed temporal structure [35]. In this case, texture discrimination depends on neurons' reporting the temporal sequence of individual events (bumps or ridges) within a whisk. An efficient encoding scheme could therefore comprise a signal based on firing rate to discriminate strongly differing textures (e.g., very rough versus very smooth), together with a signal based on temporal firing patterns to discriminate the finer details of textures within a single category of coarseness. We propose that the adaptive responses presented here could underlie this scheme by permitting clear changes in firing rate to distinguish between high- and low-variance motion signals, while enhancing the representation of local features within the context of each variance.

Adaptive gain rescaling may also aid texture discrimination by making responses to a given texture invariant to the overall magnitude of whisking. The whisking motion used to analyze objects is actively controlled by primary motor cortex, and whisking bouts can vary in amplitude and speed (reviewed in [65]). Ideally object perception would be invariant to changes in whisking scale. Adaptive gain rescaling might allow neurons to adjust their tuning not only in response to the statistical context of external events, but also in response to the whisking-related sensory signals generated by the rat itself. For example, if the sensory system adapts to high-magnitude whisking signals before object contact, the high-velocity events induced by subsequent object contact would be efficiently encoded.

## Materials and Methods

All experimental procedures were performed in accordance with animal care and use requirements of the National Institutes of Health, the European Commission, and the institution (Scuola Internazionale Superiore di Studi Avanzati). After performing pilot studies on 18 rats for selection of stimulus parameters, we collected data from six adult (∼350 g) male Wistar rats.

### Surgical methods and data collection.

Animals were anesthetized with urethane (1.5 g/kg body weight i.p.) and stabilized in a stereotaxic frame.

The technique for Utah array experiments has been described [37,66,67]. Briefly, a craniotomy was centered 2 mm posterior and 6 mm lateral to bregma. The dura was left intact. A “Utah” array consisting of a 10 × 10 grid of 1.5-mm-long electrodes with 400-μm tip-to-tip spacing (Cyberkinetics Neurotechnology Systems, http://www.cyberkineticsinc.com) was inserted into the left BC using a pneumatic impulse inserter (Cyberkinetics Neurotechnology Systems), and a reference wire was positioned. Electrode penetration depths were approximately 700–1,000 μm. Signals from all 100 electrodes were simultaneously amplified and filtered (gain, 5,000; band-pass 250–7,500 Hz [Cyberkinetics Neurotechnology Systems]). Event thresholds were set equal to 2.5–3.5 times root mean square voltage: 1-ms-duration event waveforms were extracted at 30 kHz (Cyberkinetics Neurotechnology Systems).

For Michigan probe experiments, a craniotomy of size 3–4 mm on the side, centered 2.5 mm posterior and 5.5 mm lateral to bregma, was performed. A durectomy was performed over the BC area and a four-shank, 16-channel silicon-substrate probe (University of Michigan Center for Neural Communication Technology, http://www.cnct.engin.umich.edu) was inserted using a motorized microdrive (Narishige, http://www.narishige.co.jp). Electrode depths on each shank spanned 150 μm and inter-shank spacing was 125 μm. Electrodes were advanced until the lowest was at a depth of 780–820 μm. Signals were amplified and filtered at 2,000–10,000 gain and 600–9,000 Hz band-pass (Neuralynx, http://www.neuralynx.com). Event voltage thresholds were set manually and monitored throughout the experiment. For each event, 32 data points were extracted at a 38.1-kHz sampling rate.

### Stimulus design and presentation.

During stimulation, whiskers were deflected in one dimension with a random sequence of positions whose instantaneous values were distributed according to a Gaussian [15,16]. Stimulus waveforms were generated in MATLAB (The MathWorks, http://www.mathworks.com) at a sampling rate of either 2 kHz or 4 kHz (10–20× stimulus roll-off frequency, see below). The sequence had approximately constant power over the frequency range for which BC neurons are responsive (up to 200–250 Hz), but was low-pass filtered above that range to eliminate wafer resonance effects (see below); the stimulus autocorrelation full width at half maximum was approximately 5 ms. The stimulus variance switched between two values every 5 s, so that a full stimulus cycle lasted 10 s. The low standard deviation equaled 0.7 times the high standard deviation. Since each variance defined an entire separate stimulus distribution, we used only two variance values, to ensure that neuronal spiking responses to both distributions could be well sampled within an experiment's duration. The variance was imposed on the Gaussian stimulus waveform using a mask that switched smoothly between high and low values (Figure 1A). Stimulus frequency filtering was identical for both conditions, so that both had the same frequency spectrum.

Piezoelectric wafers can have resonant frequencies in the hundreds of hertz and a transfer function dependent on mechanical configuration. To prevent distortions in wafer motion, stimuli were first low-pass filtered (∼210 Hz; see above). To verify the wafer's linearity within the remaining stimulus frequency range, we optically monitored [33,37] wafer displacements over the frequency range used in our experiments. Any small deviations from linearity were compensated for by convolving the desired stimulus with the wafer's inverse transfer function, generating the final signal fed to the amplifier. Finally, stimuli were checked with the optical sensor to ensure that wafer motion followed the desired stimulus waveform.

During the recording session, the stimulus signal was transmitted to a digital to analog board (NI 6713, National Instruments, http://www.ni.com) and from there to a custom-made amplifier (Erik Zorzin), which fed the voltage signal to a piezoelectric wafer (Morgan ElectroCeramics, http://www.morganelectroceramics.com). Several whiskers on the right side of the snout were introduced, up to a distance 2–3 mm from their base, into a glass pipette (inside diameter ∼0.8 mm) glued to the wafer. Whiskers followed pipette displacements faithfully (see Protocol S1 for a detailed description). For the high-variance stimuli, maximum displacement range was 52 μm; angular range was approximately 1.5°. Standard deviation was 4.1 μm in position and 2.5 mm/s in velocity. Maximum speed was 39 mm/s. These parameters were kept constant across all neurons: their choice did not depend on each neuron's specific tuning properties. In general, at these stimulation levels BC neurons were responsive but well under saturation.

### Neuronal waveform classification and selection.

Recording sessions lasted over 3 h, the time necessary to collect the minimum of ∼5,000 spikes required for STC analysis. Cortical activity in anesthetized rats can be nonstationary over such long periods [28]. Channels were selected for spike sorting if the unsorted, multineuron activity was stable through the over 3-h experiment and showed responses to Gaussian noise stimulation.

All data processing was carried out in MATLAB. Cluster cutting was performed automatically using either SAC software [68] (for Utah array experiments) (http://www.cyberkineticsinc.com/content/researchproducts) or the Klustakwik program by K. D. Harris (http://qneuro.rutgers.edu) executed from the MClust toolbox written by A. D. Redish [69] (for Michigan probe experiments) (http://www.cbc.umn.edu/∼redish/mclust). Clusters were manually checked and corrected if necessary, using SAC or MClust. A spike cluster was accepted as a single neuron only if its waveform shape remained consistent and stable throughout, its average firing rate was high enough for adequate sampling (>0.5 Hz), and its inter-spike interval distribution increased smoothly from a refractory period >1 ms. Responsiveness and selectivity to noise stimuli were checked by estimating covariance difference matrices (see below) and testing whether they had eigenvalues significantly different than zero. In this way, only single neurons sensitive to features of our chosen stimuli were included in the analysis. Neurons that did not meet these criteria might have been either not responsive to stimulation or else not sensitive to the chosen (vertical) direction of whisker motion [43]. Activity from a total of 37 channels underwent this selection process. A total of 34 recordings were responsive and stable and allowed measurement of rate adaptation. Ten well-isolated single neurons passed all criteria for STC analysis.

### Estimation of adaptive time courses.

We estimated a time course for each rate curve by finding the first time bin where the firing rate was less than 1/e times its peak. For rate curves well fit by a single exponential, this number is equivalent to the fit's decay time constant, while for other curves it provides a well-defined criterion for time course definition.

### Covariance analysis of stimulus–response relationships.

STC and STA analyses characterize a neuron's stimulus–response relationship by correlating the neuron's spike times with variations in the stimulus (i.e., comparing the stimuli that evoked spikes to those drawn from the random “prior” distribution of stimuli uncorrelated with spikes) (see Protocol S1 and Figure S1) [41,53]. The simplest characterization of the spike-triggered distribution is its mean, given by the STA, but if the mean of the spike-triggered distribution does not differ from that of the prior, the STA will not capture the stimulus dependence of the neuron's spike probability (Figures S1B and 3A). This situation may occur when the neuron is sensitive to features that have a symmetry or an invariance such that the mean spike-triggering stimulus is zero. However, in this case, the spike-triggered and prior distributions will nonetheless have a different structure (Figure S1) (also see [22,40]). This structure can be estimated by considering the next order statistic of the distribution, the covariance. STC analysis characterizes neuronal selectivity by picking out the directions in stimulus space along which the spike-triggered and prior distributions' variances have maximal difference. This is done by estimating the STC matrix, **C**, and the prior covariance matrix, **C** ^prior^, and then subtracting one from the other to obtain **Ĉ = C** −** C** ^prior^. Covariance differences between the spike-triggered and prior distributions appear as visible structure in this matrix (Figures S1D and 3B). Diagonalization of **Ĉ** determines an orthonormal coordinate frame in stimulus space defined by the directions of maximal change in variance [22]. Each eigenvalue indicates the change in variance between the prior and spike-triggered distributions along the particular direction in stimulus space given by the corresponding eigenvector. The stimulus dimensions or features most relevant to the neuron's response correspond to the eigenvalues with largest absolute magnitudes. For Gaussian white-noise stimuli, the eigenvectors of **Ĉ** provide a coordinate system spanning the stimulus subspace of interest; for colored noise stimuli, eigenvectors are convolved with the stimulus autocorrelation, which did not qualitatively affect our results [42].

We conducted STC analyses separately for responses in the high- and low-variance periods. First, we assembled spike-triggered stimulus distributions by collecting stimulus segments extending from a time *T*_pre_ prior to the spike to the time of the spike. Only spikes evoked during the final, steady-state part of each variance period (the interval 2–5 s after each variance switch) were considered. We then constructed and diagonalized **Ĉ**: most of its eigenvalues were near zero. To estimate the noise level due to finite sampling, and to establish which eigenvalues were significantly different than zero, we computed a confidence interval on the eigenvalue magnitudes for a neuron firing *n* spikes at random, where *n* was the actual number of spikes collected [48,57,59]. We first generated the covariance matrix **C**_chance_ by taking *n* random samples from the prior distribution, then computed the difference matrix** Ĉ**_chance_ **= C**_chance_ – **C** ^prior^_chance_, and finally diagonalized **Ĉ**_chance_ to obtain the 2.5th and 97.5th percentiles of its eigenvalue distribution; this was repeated 100 times, and thresholds for significance were set at the median 2.5th and 97.5th percentiles.

The next stage in the analysis was to determine the dependence of the neuron's spiking probability on a stimulus, 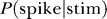 . Within the linear–nonlinear representation's framework, this dependence is completely described by the nonlinear input–output function stating how firing is modulated by the linear projections {*k_i_*}*_i_*_ = 1…*n*_ between the stimulus and the *n* significant features constituting the neuron's receptive field: 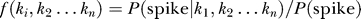 . For simplicity we limited our analysis to the one-dimensional functions describing how the stimulus projection onto each significant stimulus feature modulated the neuron's firing probability: 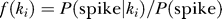 , where *k_i_* denotes the stimulus segment's projection onto the feature of interest. Using Bayes's theorem, this equation can be rewritten as 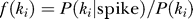 , where 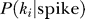 and *P*(*k_i_*) are the spike-triggered and prior distributions of stimuli projected onto the feature. We estimated each input–output function by first computing the projection *k_i_* of each stimulus segment in the spike-triggered ensemble onto the relevant feature and projecting the feature onto the entire stimulus sequence to estimate the prior. We then built histograms for each distribution and divided the results bin by bin. We applied kernel smoothing to the 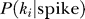 histogram; the prior formed a smooth Gaussian. Estimates were repeated for 30 different random bootstrap samples of the spike-triggered projections: error bars in the plots show the procedure's standard deviation. Estimated input–output functions had a characteristic symmetric U-shaped dependence on projection (Figure 3C and 3D), implying that neurons encoded the absolute value of the stimulus feature.

Information carried in spike times about stimulus features was computed using the method of [38] and [46]. Two quantities were computed: first, the information about the most significant feature obtained as the first eigenmode of **Ĉ**; and second, the information summed over all significant features, equivalent to the total information captured by the model assuming that all features contribute independently.

Finite sampling can lead to errors in the estimation of information [70]. We computed the histograms 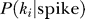 and *P*(*k_i_*) for a range of discretizations δ*k* of *k_i_*. An almost constant information plateau was generally reached for ranges of δ*k* from 0.75 to 1.5 stimulus standard deviations. Discretizing *k_i_* into 20 bins for a fixed δ*k* equal to 0.4–0.5, we also estimated the information for a series of subsamples of different sizes, *I*(*n*), where *n* equals the number of spikes in the sample. Solving for the expected behavior *I*_inf_ = *I*(*n*) – *A*/*n* [71,72] produced an information estimate *I*_inf_ that was equal to the plateau information to within our procedure's error.

Sensitivity to parameter choices was tested as follows. We usually built the spike-triggered and prior stimulus ensembles using *T*_pre_ = −150 ms, but analyses repeated for *T*_pre_ = −100 ms or −80 ms did not change final results, while *T*_pre_ shorter than −60 ms or −70 ms did alter the results for some neurons, consistent with visual inspection of **Ĉ** matrices (Figure 3B). This implies that the generation of a spike at time *t* depended on stimulus features defined over the 100 ms preceding *t*. The brief stimulus integration time (approximately 100 ms) compared to the average inter-spike interval (approximately 1 s for a typical neuron responding at ∼1 spike/s) suggests that information was carried by single spikes independently rather than by spike ensembles, so that information calculations based on single spikes were a good approximation to the spike train information.

We also repeated our analyses using features and stimulus segments binned at a range of times spanning 1–4 ms; changes in temporal resolution did not drastically alter the structure of **Ĉ**. However, the smallest time bins led to inadequate sampling of **Ĉ.** Conversely, overly large time bins smoothed over the features' temporal structure. Evaluating the information, we found a stable regime around 2 ms, which we then used for all our results.

### Simulations.

Simulations were run in MATLAB. The model consisted of an idealized neuron that, upon presentation of a stimulus segment, generated a spike with probability 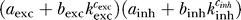 , where *k*_exc_ and *k*_inh_ represent the stimulus projections onto an excitatory and an orthogonal suppressive filter, respectively (Figure 6A shows an example): *a, b,* and *c* were free parameters that were chosen independently for the excitatory and inhibitory filters (with *b*_inh_ negative). In simulations, parameters were varied to verify the generality of the conclusions drawn from the model. When incorporated into the model, input rescaling was implemented by dividing the stimulus by its standard deviation.

## Supporting Information

Protocol S1 Notes on Stimulus Design and Application, and Full Legend for Figure S1
(54 KB DOC)

Figure S1 STA and STC Applied to a Model Neuron A full explanation is given in Protocol S1.
(A) A model neuron was stimulated with a sequence of whisker displacements (A1). Responses were analyzed in two conditions. Condition 1: the neuron fired when a large change in displacement across successive time steps was positive (A2). Condition 2: the neuron fired for absolute changes in displacement (A3).
(B) Geometrical representation of STA and STC. (B1) A sample of the overall, or “prior,” stimulus distribution. (B2) Overall stimulus sample as in B1 overlaid with spike-triggering events under condition 1. (B3) Overall stimulus sample overlaid with spike-triggering events under condition 2.
(C) STA on stimulus waveforms. (C1) STA waveforms for condition 1.** (**C1A) STA plotted in terms of position. (C1B) STA computed in “velocity space.” (C2) STA for condition 2. (C2A) STA computed in “position space”; positive and negative changes in displacement cancel out (see A3). (C2B) STA in “velocity space.” (C2C) STA in “absolute velocity,” or “speed,” space. Taking the absolute value of the velocity waveform gives only positive values, resulting in a positive STA.
(D) Covariance matrices for prior and spike-triggered distributions in “position space.” Color bar applies to all panels. (D1) Covariance matrix of the prior distribution sampled at arbitrary pairs of points. (D2) Covariance difference matrix for condition 1. (D3) Covariance difference matrix for condition 2.
(5.3 MB TIF)

Figure S2 Uniqueness of the Nonadapting Neuron The plot shows information captured by neurons' STA characterization divided by information captured by their STC characterization. Values of this “information ratio” greater than one signify a “simple” receptive field well captured by the STA; values less than one signify a “complex” receptive field for which STC characterization is necessary. Information calculations are described in the main text. Left: information ratio for every recorded single neuron. Each point corresponds to a neuron. Filled gray square, nonadapting neuron (*n* = 1); filled black circles, adapting neurons from our dataset (*n* = 8) (main text); open diamonds, neurons from a separate cortical dataset acquired under the same experimental conditions (*n* = 11) (R. S. P. and M. A. Montemurro, unpublished data). Right: histogram of information ratio values for all neurons shown in the left panel. The nonadapting neuron's value was the only one above one, denoting that it was unique in that its receptive field was well captured by an STA description. Other neurons had a qualitatively different and more complex receptive field structure.
(231 KB TIF)

## Abbreviations

BC: barrel cortex
STA: spike-triggered average
STC: spike-triggered covariance
