# Supplementary material for: Shifts in Coding Properties and Maintenance of Information Transmission during Adaptation in Barrel Cortex
Source: PLoS Biol. 2007 Jan 23;5(2):e19. doi: 10.1371/journal.pbio.0050019 (PMC1779810; doi:10.1371/journal.pbio.0050019)
Supplement: Supplementary file 1 [file pbio.0050019.sd001.doc]

**Maravall et al., Shifts in Coding Properties and Maintenance of Information Transmission during Adaptation in Barrel Cortex**

**Protocol S1**

**Notes on stimulus design and application**

*Non-separability of stimulus distribution mean and variance*

To obtain unbiased, easily interpretable results from STC analysis, stimuli were drawn from Gaussian distributions (for further explanation, see [1-3]). For neurons sensitive to stimulus position or velocity, changes in the Gaussian distribution’s variance would not affect its mean; however, neurons sensitive to speed (velocity absolute value) could process changes in variance as changes in mean. This is because the mean and variance of the distribution of absolute values are not independent. Ideally, variance could have been isolated as a stimulus feature by adding specific waveforms to the low-variance stimulus, thus making its mean speed equal to that of the high-variance stimulus. However, limitations inherent to mechanical stimulation devices prevent this. Adding a constant velocity to the stimulator’s motion would make it “wander away” from the stimulus site very soon. Adding discontinuous stimuli (such as a velocity that switched sign every time the wafer’s range limits were reached) would cause the wafer to resonate and make response analysis intractable; further, discontinuities would be more frequent in high-variance than in low-variance periods. We recorded responses to modified stimuli, adding slowly varying waveforms (e.g. a slow sine wave) to the basic Gaussian values, but neurons were effectively insensitive to this stimulus component unless its frequency was high enough to distort the basic waveform too severely for STC analysis. The remaining possibility for isolating absolute velocity mean and variance would be to change the stimulus temporal autocorrelation or frequency spectrum, since access to higher frequencies implies access to higher velocities. However, this would produce a serious confound, since it would not be possible to separate adaptation to variance from adaptation to stimulus correlations (e.g. [3]).

*Stimulation configuration and verification of whisker motion*

For every electrode that acquired the firing of a stable neuronal cluster, we identified the principal whisker [4] using manual stimulation. Principal whiskers for the 10 neurons studied with STC included C2, D2, and D3. Whiskers selected for stimulation included principal whiskers for all stable clusters (2-5 in total) and were clipped to ~10 mm; the others were clipped flush to the pad.

The piezoelectric wafer-capillary tube assembly was rigidly mounted on a manual micromanipulator (E.S.F. Electronic, Göttingen, Germany). The device was positioned at a point that allowed us to guide whiskers into the capillary tube. The proximal end of the tube was about 7 mm from the skin. Visualizing the whisker pad under an operating microscope, we inserted the whiskers with a fine forceps and then advanced the tube about 4-5 mm closer to the skin and about 1 mm laterally. This ensured that all whiskers contacted the edge of the tube opening throughout the stimulator’s motion range. We checked that whisker contact with the tube was stable by gently pushing with fine forceps. When the whiskers were immobile, cortical neurons never showed position-dependent steady state responses.

Next, we verified that the whiskers could follow stimulator motion in a smooth, continuous manner by applying small lateral movements with the micromanipulator while observing through the microscope. During the subsequent recording session, stimulator motion covered a small angle (~1.5º) and therefore did not greatly perturb the whiskers’ baseline angular setting, making deviations from linearity and stick-slip movements unlikely. In previous work, we have optically monitored the whiskers under conditions similar to the present ones and found that they faithfully follow stimulator motion [5,6]. At the end of stimulation periods lasting over 3 hrs, whiskers remained in the initial position relative to the capillary tube, confirming the stability of the stimulator and the tube-whisker interface.

We also performed post-hoc checks on whisker trajectory by testing how measures of stimulus encoding were affected when temporal jitter or noise were artificially added during the analysis phase. Varying the relationship of spike times to stimulus waveform by a small factor (4 ms/min) caused a complete loss of stimulus-response structure, as measured both by cyclic variations in spike rate and by the STC analysis. Because such structure existed only if data were analyzed according to the nominal stimulus waveform, the neuronal responses must have had a precise temporal relationship to whisker movement. If the mechanical link between stimulator and whiskers had introduced any systematic jitter or phase lag, we could not have recovered the stimulus-response relationships of Figs. 4-5, and this is another indication that whisker movement was an accurate copy of stimulator movement. If any mechanical imprecision in the stimulus were undetected, its effect would be a minor “washing out” of tuning functions, a decrease in their signal-to-noise ratio, and a slight reduction in information transmission rates. The fundamental results concerning the role of adaptation in stimulus coding would not be altered.

**Full legend for Figure S1, Spike-triggered averaging (STA) and spike-triggered covariance (STC) applied to a model neuron**

**A**: A model neuron was stimulated with a sequence of fluctuating whisker displacements, distributed as a Gaussian with unit standard deviation (**A1**: sequence for 50 time steps). Responses were analyzed in two conditions. *Condition 1*: the neuron fired when the change in displacement across successive time steps was ***positive***, greater than 1.6 (**A2**; spike-evoking events in magenta). *Condition 2*: the neuron had the same threshold but fired for ***absolute*** (positive or negative) changes in displacement (**A3**; spike-evoking events in green). **B**: Geometrical representation of STA and STC. **B1**: A random sample of the overall or “prior” stimulus distribution: each dot represents displacement at one time step against displacement one time step later. **B2**: Overall stimulus sample as in B1 overlaid with spike-triggering events under *condition 1* (magenta). The spike-triggering distribution is a subset of the prior distribution. The shift in the mean of the spike-triggering distribution with respect to the mean of the prior is captured by the STA (yellow line). The STA specifies the spatial dimension of the shift between distributions. In this example, the dimension is velocity (displacement between successive time steps). The STA uncovers one dimension *at most*, therefore it is useful only for neurons selective for a single stimulus feature. **B3**: Overall stimulus sample overlaid with spike-triggering events under *condition 2* (green). Here, the difference between the prior and the spike-triggered distribution is not captured by the STA because the mean (yellow cross) has not shifted. Instead, STC identifies the relevant stimulus features as the directions along which the spike-triggered distribution’s covariance differs from that of the prior. In this example, the dimension picked out by STC is, again, stimulus velocity. However, two symmetric regions along this dimension now trigger spikes: the neuron is sensitive to speed, the absolute value of velocity. In general, STC analysis extracts a “subspace” containing the stimulus features (dimensions) that the neuron is sensitive to. STC can extract any number of features, overcoming a limitation of STA. Having extracted the relevant feature or dimension, the neuron’s response to a stimulus event can be predicted by computing the stimulus projection onto the dimension, or dot product of the dimension and the vector from the origin to the stimulus point. Here, if the projection is greater than 1.6, a spike is fired. In general, responses will be determined by stimulus projections onto all features of interest. **C**: STA on stimulus waveforms. **C1**: STA waveforms for *condition 1*. **C1A**: The STA plotted in terms of position corresponds to a whisker displacement from a value below to a value above the prior average. The neuron fired with some latency after the relevant event, so the STA waveform is at time steps -2 and -1 relative to spike time. This biphasic structure is suggestive of a temporal differential, the derivative of displacement (velocity). **C1B**: Red line, STA computed in “velocity space”, i.e. on the differential of the displacement waveforms in A. Magenta crosses, result of differentiating the “position space” STA calculated in C1A. The crosses fall exactly on the red line, meaning that “position space” and “velocity space” analyses are equivalent. **C2**: STA for *condition 2*. **C2A**: The STA computed in “position space” is negligible because positive and negative changes in displacement cancel out (see A3). **C2B**: The STA in “velocity space” (dark green line) is also negligible. Green crosses, differentiated “position space” STA shown in C2A. “Position space” and “velocity space” analyses are equivalent. **C2C**: STA computed in “absolute velocity” or “speed” space. Taking the absolute value of the velocity waveform gives only positive values, resulting in a positive STA. If a neuron is sensitive only to velocity, the STA computed in “speed space” gives similar information to the STC computed in the original stimulus space; however, STA can recover only a single feature while STC can recover multiple features. **D**: Covariance matrices for prior and spike-triggered distributions in “position space”. Color bar applies to all panels. **D1**: Covariance matrix of the prior distribution sampled at arbitrary pairs of points. The plot shows covariance values for the displacement at an arbitrary time (defined as the origin, 0) paired with displacements at neighboring times (-4, -3, -2, -1, 0, 1 relative to the origin). Thus, the value at entry (-1,-1) is the variance at time -1; since the origin is arbitrary, all points on the diagonal are equivalent except for sampling noise. **D2**: Covariance difference matrix for *condition 1*. The plot shows the difference between the STC matrix and the prior covariance matrix from D1. Spike time is 0. Hot spots denote large positive differences relative to the prior covariance matrix; cold spots denote large negative differences. For instance, diagonal hot spots signify times at which the variance of the spike-triggered distribution (magenta “cloud” in B2) was much larger than the variance of the prior. **D3**: Covariance difference matrix for *condition 2*: difference between the STC matrix and the prior covariance matrix from D1. Although here STA fails to identify any structure (C2), STC reveals significant structure. Significant stimulus dimensions or features can be extracted from these matrices using standard linear algebra diagonalization techniques.

1. Simoncelli EP, Pillow JW, Paninski L, Schwartz O (2004) Characterization of neural responses with stochastic stimuli. In: Gazzaniga M, editor. The Cognitive Neurosciences. Cambridge, MA: MIT Press. pp. 327-338.

2. Bialek W, De Ruyter Van Steveninck RR (2005) Features and dimensions: motion estimation in fly vision. Preprint, lanl.arxiv.org:q-bio.NC/0505003.

3. Sharpee TO, Sugihara H, Kurgansky AV, Rebrik SP, Stryker MP, et al. (2006) Adaptive filtering enhances information transmission in visual cortex. Nature 439: 936-942.

4. Simons DJ (1978) Response properties of vibrissa units in rat SI somatosensory neocortex. J Neurophysiol 41: 798-820.

5. Arabzadeh E, Petersen RS, Diamond ME (2003) Encoding of whisker vibration by rat barrel cortex neurons: implications for texture discrimination. J Neurosci 23: 9146-9154.

6. Arabzadeh E, Zorzin E, Diamond ME (2005) Neuronal encoding of texture in the whisker sensory pathway. PLoS Biol 3: e17.
